# Mangelernährung in der Inneren Medizin: Screening, Assessment und Bedeutung

**DOI:** 10.1007/s00108-023-01525-x

**Published:** 2023-05-22

**Authors:** Nina Kaegi-Braun, Carla Gressies, Pascal Tribolet, Franziska Stumpf, Bettina Keller, Philipp Schuetz

**Affiliations:** Innere Medizin, Medizinische Universitätsklinik, Kantonsspital Aarau, Tellstrasse 25, 5001 Aarau, Schweiz

**Keywords:** Ernährungstherapie, Mangelernährung/diagnostische Kriterien, Mangelernährung/Biomarker, Personalisierte Ernährung, Schweiz, Nutrition therapy, Malnutrition/diagnostic criteria, Malnutrition/biomarkers, Personalized nutrition, Switzerland

## Abstract

Krankheitsbedingte Mangelernährung hat starken Einfluss auf den weiteren Krankheitsverlauf und die Sterblichkeit, insbesondere bei chronisch kranken Patient*innen. In den letzten Jahren konnte in großen randomisierten Studien gezeigt werden, dass eine individuelle Ernährungstherapie das klinische Outcome von medizinischen Patient*innen mit einem Risiko für Mangelernährung im Krankenhaus und in der Nachsorge signifikant und relevant verbessert. Angesichts des steigenden Anteils multimorbider Patient*innen rücken deshalb die Mangelernährung sowie ihre Therapie in der Praxis und Forschung zunehmend in den Fokus. Ernährungsmedizin sollte heutzutage als effektiver und integraler Bestandteil einer ganzheitlichen Therapie in der Inneren Medizin betrachtet werden. Weitere Forschung ist aber notwendig, um neue Ernährungsbiomarker zu untersuchen und eine evidenzbasierte, personalisierte Ernährungsmedizin noch besser in den klinischen Alltag integrieren zu können.

## Abstract (en)

Disease-related malnutrition has a strong influence on the further course of the disease and mortality, especially in chronically ill patients. In recent years it could be shown in large randomized studies that an individual nutrition therapy could significantly and relevantly improve the clinical outcome of patients in internal medicine with a risk of malnutrition, both in hospital and in aftercare. Therefore, due to the increasing proportion of multimorbid patients the significance of malnutrition and its treatment is becoming increasingly more important in the practice and in research. Nutritional medicine should nowadays be considered as an effective and integral component of a holistic treatment in internal medicine; however, further research is necessary in order to investigate new nutritional biomarkers and for a better integration of an evidence-based personalized nutritional medicine into routine clinical practice.

## Definition – was ist unter Mangelernährung zu verstehen?

**Figure 1:**
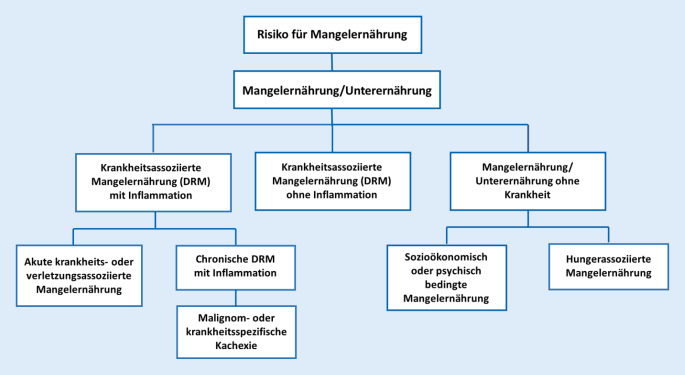

Nach wie vor existiert keine einheitliche Definition für den Begriff „Mangelernährung“. Die Deutsche Gesellschaft für Ernährung (DGE) definiert Mangelernährung in ihrem Klinikleitfaden [1] als einen „Zustand, der aus einer mangelnden Zufuhr oder Aufnahme von Energie und Nährstoffen über die Nahrung entsteht, zu einer veränderten Körperzusammensetzung führt und mit messbaren Veränderungen körperlicher und mentaler Funktionen verbunden ist. Als Folge verschlechtern sich die Prognose und der klinische Krankheitsverlauf.“ Diese Definition deckt sich weitestgehend mit jener der European Society for Clinical Nutrition and Metabolism (ESPEN; [2]). Die ESPEN unterteilt Mangelernährung ätiologisch in drei Hauptgruppen (Abb. 1): die krankheitsassoziierte Mangelernährung („disease-related malnutrition“ [DRM]) mit oder ohne Inflammation und die Mangelernährung ohne Krankheit. Im klinischen Kontext der westlichen Industrieländer spielt die nichtkrankheitsassoziierte Mangelernährung eine untergeordnete Rolle, weshalb hier vor allem auf die Formen der DRM eingegangen wird.

Der Begriff „Mangelernährung“ war lange nicht einheitlich definiert

Die DRM mit Inflammation lässt sich wiederum in eine akute sowie eine chronische Form unterteilen. Die Kachexie bildet hier eine Untergruppe. Ihnen gemeinsam ist die inflammatorische Komponente, die unter anderem zu Anorexie, Gewichtsverlust sowie Sarkopenie führt [3]. Während bei der chronischen Form meist langwierige Krankheitsverläufe mit eher milder Inflammation vorliegen, betrifft die akute Form meist Schwerkranke oder sogar Intensivpatient*innen, die einen weit ausgeprägteren Stressmetabolismus aufweisen [4].

## Welche Rolle spielt die Mangelernährung bei uns?

**Figure 2:**
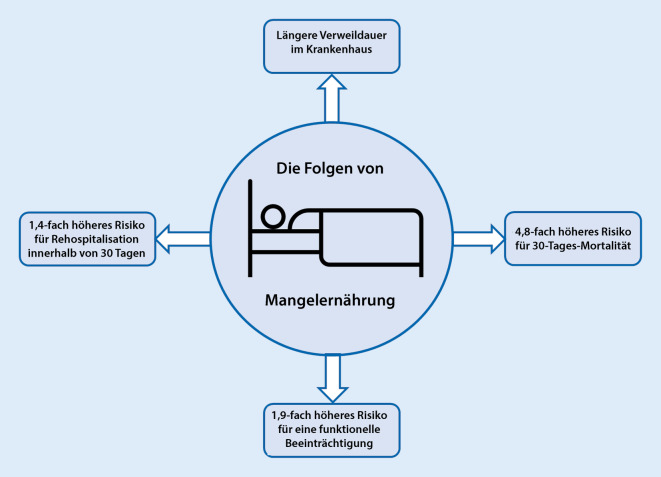

Gemäß der Literatur liegt der Anteil mangelernährter Patient*innen im Krankenhaus bei etwa 30 % [5, 6]. Bei älteren Personen mit medizinischer Betreuung zu Hause liegt die Mangelernährungsrate gemäß niederländischen Zahlen ähnlich hoch [7]. Die Entstehung von Mangelernährung ist verschiedenen Faktoren geschuldet. Zum einen gibt es viele sozioökonomische Aspekte wie das Alleinsein, ein geringes Einkommen oder ein niedriges Bildungsniveau [8], die eine Mangelernährung begünstigen. Zum anderen spielen besonders bei der DRM auch metabolisch-biochemische Prozesse eine wichtige Rolle bei der Entstehung von Mangelernährung. Inflammatorische Prozesse führen beispielsweise zu verringertem Appetit [9] oder besonders bei akut erkrankten Patient*innen zu einem ausgeprägten Stressmetabolismus. Dieser führt durch den Abbau von Protein und im Verlauf auch von Fett zu Gewichtsverlust und – verstärkt durch Immobilisation – auch zu Sarkopenie [10]. Zudem treten eine periphere Insulinresistenz sowie eine Veränderung der Hypothalamus-Hypophysen-Achse mit einer vermehrten Ausschüttung von Stresshormonen wie Kortisol auf. Im chronischen Verlauf kommt es zudem zu einer peripheren Resistenz gegenüber weiteren stoffwechselrelevanten Hormonen wie Schilddrüsen- und Wachstumshormonen [4]. Diese Prozesse führen insgesamt zu einer katabolen Stoffwechsellage. Als Folgen davon zeigen sich bei Mangelernährten eine erhöhte 30-Tages-Mortalität, eine verlängerte Krankenhausliegedauer und eine erhöhte 30-Tages-Rehospitalisierungsrate, zudem ein Verlust an Lebensqualität, einhergehend mit einem höheren Risiko für Funktionseinschränkungen (Abb. 2, [11]). Es handelt sich insgesamt also um ein weitverbreitetes Problem mit weitreichenden Konsequenzen sowohl für die Patient*innen als auch für das Gesundheitssystem als Ganzes.

## Diagnostik

### Diagnostische Tools

**Figure 3:**
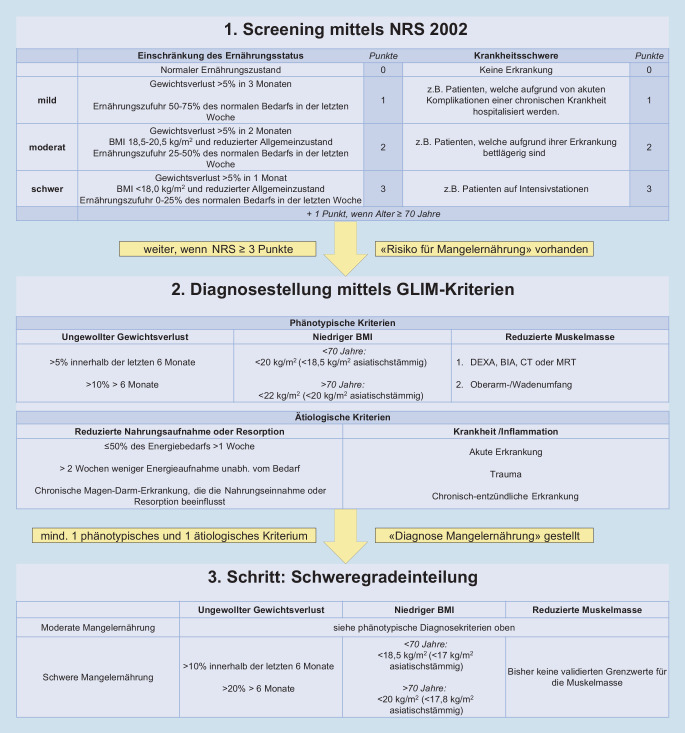

Für die Erkennung einer Mangelernährungssituation stehen diverse Screening- und Assessment-Tools zur Verfügung. Im stationären Setting häufig angewendet wird beispielsweise das Nutritional Risk Screening (NRS 2002), das anhand von Ernährungssituation, Krankheitsschwere und Alter einen Punktescore generiert und das Risiko für eine Mangelernährung abschätzt ([12]; siehe auch Abb. 3). Andere Screening-Tools beruhen oft auf ähnlichen Parametern und sind für unterschiedliche Settings validiert [13]. Nach dem Screening erfolgt meist ein genaueres Assessment mit detaillierter Ernährungsanamnese, anthropometrischen und funktionellen Messungen, Parametern der Körperzusammensetzung und zum Teil auch Biomarkern.

### Neueste Fortschritte in der Diagnostik

Im Jahr 2019 wurde von der Expertengruppe Global Leadership Initiative on Malnutrition (GLIM) ein Vorschlag veröffentlicht, der zum Ziel hat, die Diagnostik der Mangelernährung international zu standardisieren [14]. Seither werden die sogenannten GLIM-Kriterien für die Diagnostik der Mangelernährung empfohlen. In Abb. 3 sind die einzelnen Kriterien gezeigt. Nach einem Screening mit einem etablierten Tool wie NRS 2002 werden phänotypische und ätiologische Kriterien erhoben; bei Vorliegen mindestens eines phänotypischen und eines ätiologischen Kriteriums kann die Diagnose der Mangelernährung gestellt werden. Anschließend wird anhand der phänotypischen Kriterien eine Schweregradeinteilung vorgenommen.

### Erfassung der Diagnose im System der „Diagnosis Related Groups“ (DRG)

Während die GLIM-Kriterien einen Leitfaden für die klinische Tätigkeit darstellen, sieht es bei der Codierung des Krankheitsbilds noch uneinheitlich aus. In der Schweiz wird aktuell das NRS 2002 als Grundlage für die Codierung und finanzielle Vergütung verwendet, während es im internationalen Codierungssystem der Internationalen statistischen Klassifikation der Krankheiten und verwandter Gesundheitsprobleme (ICD-11) weiterhin lediglich einen Code für „undernutrition in adults“ gibt, der einzig durch einen Body-Mass-Index ≤ 18,5 kg/m^2^ definiert ist [15]. Es wird jedoch von verschiedenen Ernährungsgesellschaften eine Anpassung der aktuellen Codierungsregeln unter Einsatz der GLIM-Kriterien gefordert [16].

## Therapie

### Ist eine Ernährungstherapie sinnvoll?

Lange blieb die Frage offen, ob die schlechtere Krankheitsprognose bei Mangelernährten reversibel ist bzw. mit einer Ernährungstherapie verbessert werden könnte. In den letzten Jahren konnten diesbezüglich neue wissenschaftliche Erkenntnisse gewonnen werden. Diese wurden 2019 in einer Metaanalyse von Gomes et al. [17] zusammengefasst. Die zwei größten randomisierten, kontrollierten Studien (RCT) waren die NOURISH-Studie (Nutrition effect On Unplanned ReadmIssions and Survival in Hospitalized patients) und die EFFORT-Studie (Effect of Early Nutritional Therapy on Frailty, Functional Outcomes and Recovery of Undernourished Medical Inpatients Trial). Die NOURISH-Studie untersuchte den Effekt einer High-protein-Trinknahrung mit β‑Hydroxy-β-Methylbutyrat (HMB) bei 652 älteren hospitalisierten Patient*innen im internistischen Bereich im Vergleich zu einem Placebo. Diese Arbeit konnte zeigen, dass die 90-Tages-Mortalität in der Interventionsgruppe signifikant geringer war als in der Kontrollgruppe (4,8 % vs. 9,7 %; relatives Risiko 0,49; 95 %-Konfidenzintervall [KI] 0,27–0,90; *p* = 0,018; [18]). In der multizentrischen Schweizer EFFORT-Studie [19] wurde bei insgesamt 2088 Patient*innen der Nutzen einer frühen individuellen Ernährungstherapie durch eine Ernährungsberater*in im Vergleich zur Standardkrankenhausernährung untersucht. Diese Studie zeigte eindrücklich, dass individuelle Ernährungsstrategien zur Erreichung der Energie- und Proteinziele das Risiko schwerer Komplikationen (23 % vs. 27 %; Odds Ratio [OR] 0,79; 95 %-KI 0,64–0,79; *p* = 0,023) und die 30-Tages-Mortalität (7 % vs. 9 %; OR 0,65; 95 %-KI 0,47–0,91; *p* = 0,011) signifikant reduzieren konnten. Ebenfalls führte die individuelle Ernährungstherapie zu einer Verbesserung der Funktionalität und Lebensqualität.

### Versorgung mangelernährter Patient*innen im Krankenhaus

**Figure 4:**
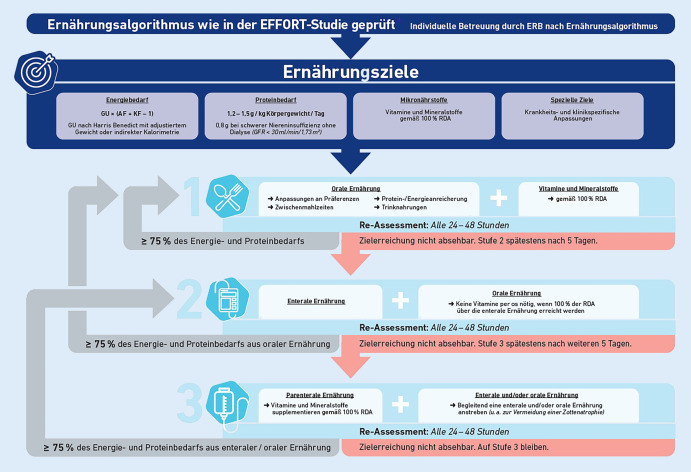

Obwohl die Studien in den oben erwähnten Metaanalysen nicht das gleiche ernährungstherapeutische Vorgehen hatten, ähnelten sich die Konzepte und entsprachen weitestgehend den jüngsten Empfehlungen der ESPEN [20]. Ein mögliches Vorgehen, wie es auch in der EFFORT-Studie geprüft wurde, ist in Abb. 4 dargestellt. Die Energie- und Proteinziele werden dabei für jede Patient*in individuell bestimmt. Bei der Energiebedarfsberechnung werden neben dem Grundumsatz auch die Erkrankung und die Aktivität des Individuums berücksichtigt. Der Grundumsatz kann beispielsweise mit der indirekten Kalorimetrie oder anhand einer validierten Formel, etwa der Harris-Benedict-Formel, bestimmt werden, wobei bei adipösen Personen das adjustierte Gewicht verwendet wird [21, 22].

Die enterale Ernährung wird der parenteralen vorgezogen

Die tägliche Proteinmenge sollte zwischen 1,2 und 1,5 g/kgKG liegen, wobei bei Patient*innen mit Niereninsuffizienz von 0,8 g/kgKG ausgegangen wird [19]. Vitamine und Mineralstoffe können routinemäßig supplementiert werden, wobei diesbezüglich die Evidenz noch unklar ist [23]. Anhand einer initial oralen Ernährung sollte versucht werden, die individuell festgelegten Ziele zu erreichen, mindestens jedoch 75 % davon. Werden die Ziele nicht zu mindestens 75 % durch orale Maßnahmen erreicht, sollte nach maximal 5 weiteren Tagen eine enterale oder parenterale Ernährung in Betracht gezogen werden. Dabei ist zu beachten, dass die enterale Ernährung der parenteralen vorgezogen wird.

### Vorgehen nach Entlassung aus dem Krankenhaus

Wie zuvor beschrieben, gibt es mittlerweile starke Evidenz für eine Ernährungstherapie im Krankenhaus. Weiterhin besteht jedoch eine Forschungslücke für die Weiterführung dieser Therapie nach Entlassung aus dem Krankenhaus und allgemein im ambulanten Sektor. Dies zeigt sich auch daran, dass Empfehlungen in den aktuellen Leitlinien der Fachgesellschaften wie beispielsweise der ESPEN größtenteils fehlen.

Eine Sekundäranalyse der oben erwähnten EFFORT-Studie zeigte jedoch auf, dass eine Weiterführung der Ernährungstherapie womöglich notwendig ist. Während nach 30 Tagen ein Überlebensvorteil in der Interventionsgruppe bestand, verschwand dieser Effekt beim 180-Tages-Follow-up und die Mortalitätsraten der Gruppen glichen sich an (Interventionsgruppe: 23,2 %; Kontrollgruppe: 24,6 %; [24]). Dies legt die Hypothese nahe, dass ein adäquates Ernährungsmanagement auch nach Krankenhausentlassung notwendig ist, um einen Langzeiteffekt hinsichtlich Mortalität und Morbidität zu erreichen. Um diese Hypothese zu testen und die Forschungslücke zu schließen, wird aktuell an 10 Schweizer Krankenhäusern die EFFORT-II-Studie (Effect of Continued Nutritional Support at Hospital Discharge on Mortality, Frailty, Functional Outcomes and Recovery Trial) durchgeführt [15]. Mit einer geplanten Patientenzahl von über 800 Personen wird dies die größte ambulante Ernährungsstudie in diesem Bereich weltweit.

Eine kürzlich durchgeführte Metaanalyse von Kaegi-Braun et al. [25] aus dem Jahr 2022 deutet zwar darauf hin, dass eine langfristige ambulante Ernährungstherapie einen Nutzen in Bezug auf verschiedene klinische Outcomes haben kann. Diese Ergebnisse beruhen jedoch größtenteils auf heterogenen Studien mit relativ kleinen Patientenpopulationen. Die Studie schloss insgesamt 14 RCT mit einer Teilnehmerzahl von insgesamt 2438 Personen in die Analyse ein, wobei 13 Studien den Endpunkt der Langzeitmortalität untersuchten. Im Vergleich zur Kontrollgruppe hatten Patient*innen mit einer ambulanten Ernährungstherapie eine signifikant niedrigere Mortalität (10,2 % vs. 14,6 %; OR 0,63; 95 %-KI 0,48–0,84; *p* = 0,001). Ebenfalls war die Ernährungstherapie assoziiert mit einer höheren Energie- und Proteinaufnahme sowie Gewichtszunahme.

## Bedeutung

### Ernährungsmedizin – früher und heute

Die Ernährung als „Heilmittel“ ist keine neue Idee, findet sie sich doch bereits bei Hippokrates von Kos (460–377 v. u. Z.): „Let food be thy medicine and medicine be thy food“ (auf Deutsch: „Deine Nahrung sei deine Medizin und deine Medizin sei deine Nahrung“; [26]). Ernährungsprobleme wie die DRM zu therapieren, um dadurch auch den Verlauf der Erkrankung positiv zu beeinflussen, erscheint als eine intuitive und logische Schlussfolgerung. In der modernen Medizin und klinischen Forschung wurde der Ernährung als medizinischem Zweig jedoch lange wenig Bedeutung zugemessen [27].

In den vergangenen zwei Jahrzehnten erfuhr die ernährungsmedizinische Forschung jedoch einen Aufschwung – die Zahl wissenschaftlicher Publikationen zu dem Thema hat sich vervielfacht^1^. Zahlen zur Prävalenz von Mangelernährung stammen aus größeren Studien vom Anfang des 21. Jahrhunderts, wie der German Hospital Malnutrition Study [5], und wurden seitdem mehrfach bestätigt. Beispielhaft ist der weltweite *nutritionDay*, eine jährliche systematische Querschnittserhebung der Ernährungsversorgung in Krankenhäusern und Pflegeheimen mit dem Ziel, das Wissen und das Bewusstsein für Mangelernährung und die Qualität der Ernährungsversorgung zu verbessern. Auch zur Therapie von Mangelernährung werden, wie bereits erwähnt, vermehrt größere und qualitativ hochwertige Studien durchgeführt [17–19].

Mangelernährungsscreenings werden inzwischen zunehmend in Krankenhäusern implementiert

Empfehlungen in medizinischen Leitlinien, die primär auf Expertenmeinungen basierten, werden in der Folge zunehmend durch evidenzbasierte Schlussfolgerungen abgelöst [20, 28, 29]. Die empfohlenen Mangelernährungsscreenings werden inzwischen zunehmend in Krankenhäusern implementiert, Systeme für Ernährungsmanagement werden entwickelt und Ernährungstherapie zur klinischen Routine [30].

### Aktuelle Situation in schweizerischen Krankenhäusern

Eine schweizweite Kohortenstudie mit Daten des Bundesamts für Statistik untersuchte kürzlich die praktische Umsetzung der Ernährungstherapie in den Jahren 2013–2018 [30]. Es wurden 114.264 internistische Hospitalisationen identifiziert, bei denen ein Mangelernährungsrisiko diagnostiziert und codiert wurde. In 67,8 % der Fälle wurde eine Ernährungstherapie durchgeführt. Die Sterblichkeitsrate im Krankenhaus war bei mangelernährten Patient*innen mit Ernährungstherapie signifikant um 21 % reduziert. Diese Daten aus dem „realen klinischen Alltag“ stehen im Einklang mit den Ergebnissen aus den bereits erwähnten RCT. Darüber hinaus war in der Kohortenstudie die Krankenhauswiederaufnahme innerhalb von 30 Tagen nach Entlassung bei den Patient*innen mit Ernährungstherapie um 15 % reduziert. Neben diesem verminderten „Drehtüreffekt“ [31] war die Entlassung in Pflegeeinrichtungen um 11 % reduziert [30].

## Herausforderungen für die Zukunft

Obwohl die ernährungsmedizinische Forschung und die daraus entstehenden Evidenzen und Konsequenzen stark gewachsen sind, bleiben noch einige Herausforderungen zu meistern.

### Personalisierte Ernährung

Mittlerweile wurde erkannt, dass eine Ernährungstherapie nicht bei jeder Patient*in die gleiche Wirkung hat und dass sich das Therapieansprechen durchaus stark unterscheidet. Welche patienten- oder krankheitsspezifischen Faktoren für diese Unterschiede verantwortlich sind, ist derzeit noch unklar [13]. Es gibt verschiedene Ansätze, um die Idee der personalisierten Ernährung in das Ernährungsmanagement einzubringen.

So kann beispielsweise die zugrunde liegende Erkrankung einen Einfluss auf das Therapieansprechen haben. In der EFFORT-Population konnte gezeigt werden, dass Patient*innen mit einer eingeschränkten Nierenfunktion umso besser auf die Ernährungstherapie ansprachen, je niedriger die errechnete glomeruläre Filtrationsrate war [32]. Auch andere Studien an spezifischen medizinischen Populationen wie bei Patient*innen mit Herzinsuffizienz zeigten positivere Effekte durch Ernährungstherapie als in gemischten medizinischen Populationen [18, 33]. Aber auch krankheitsübergreifende Konditionen beeinflussen das Therapieansprechen grundlegend: Eine weitere Subanalyse ergab, dass eine geringe Faustschlusskraft sehr gut das Therapieansprechen voraussagen kann. Außerdem konnte in einer Arbeit gezeigt werden, dass eine Stratifizierung anhand des C‑reaktiven Proteins (CRP) als Entzündungsmarker sinnvoll sein kann. Während Patient*innen mit starker Entzündung und CRP-Leveln über 100 mg/dl keinen Überlebensvorteil durch die Ernährungstherapie hatten, profitierten Patient*innen mit geringerer Entzündung deutlich [34]. Dies könnte eine mögliche Erklärung sein, warum Ernährungsstudien, die Intensivstationspopulationen untersuchten, oftmals keinen signifikanten positiven Effekt feststellen konnten und sogar teilweise ein höheres Risiko an metabolischen Nebenwirkungen zeigten [35].

Biomarker tragen potenziell dazu bei, die Ernährungstherapie zu personalisieren

Diese und weitere Biomarker können nicht nur das Therapieansprechen vorhersagen, sondern tragen potenziell dazu bei, die Ernährungstherapie zu personalisieren. Spricht eine Patientengruppe mit bestimmten Biomarkern, etwa mit einem hohen CRP, nicht auf bisherige bzw. herkömmliche Ernährungsinterventionen an, müssen für diese neue, personalisierte Ansätze gefunden werden. Patient*innen können dann im Rahmen einer personalisierten Medizin anhand der Biomarker in Subgruppen eingeteilt und entsprechend ihren Bedürfnissen behandelt werden. So gibt es bereits spezielle Empfehlungen für Patient*innen mit Niereninsuffizienz (tägliches Proteinziel: 0,8 g/kgKG) und für intensivmedizinische Patienten.

Neben Krankheitsfaktoren können auch patientenspezifische Faktoren wie das Alter und die Genetik, aber auch sozioökonomische Unterschiede eine Rolle spielen [13]. Eine weitere Frage ist, in welchen Settings welche Ernährungstherapie sinnvoll ist. Die stärkste Evidenz besteht für die Therapie im stationären Setting, aber auch ambulant oder in Pflegeeinrichtungen sollten evidenzbasierte Standards entwickelt werden.

Mit diesen verschiedenen krankheitsspezifischen, laborchemischen, funktionellen und patientenspezifischen Faktoren könnte die Ernährungsdiagnostik verfeinert und das Ernährungsmanagement verbessert werden. Das Ziel ist ein optimales Ansprechen der Patient*innen auf eine individuelle Ernährungstherapie. Hierfür sind jedoch weitere Studien und dann gegebenenfalls eine Anpassung der aktuellen Definitionen, Diagnosekriterien und Therapieleitlinien notwendig.

### Stärkung der Ernährungsforschung

Neben den Herausforderungen einer personalisierten Ernährung gibt es auch methodische Schwierigkeiten in der ernährungsmedizinischen Forschung. Trotz der neuen GLIM-Kriterien stehen für das Feststellen einer Mangelernährung und für das Beurteilen des Ernährungszustands diverse Tools und Vorgehensweisen zur Verfügung, was die Vergleichbarkeit der Studien erschwert. Zusätzlich bestehen bei der Ernährungstherapie Unterschiede im Zeitpunkt, der Darreichungsform sowie der Menge der einzelnen Inhaltsstoffe, was ebenfalls zu einer erschwerten Vergleichbarkeit der Studien führt. Eine zunehmende Optimierung der Diagnosekriterien und klinischen Leitlinien kann helfen, Studien besser zu standardisieren. Ein weiterer (wissenschaftlich betrachteter) Nachteil ist, dass es oft unmöglich ist, die Studienteilnehmenden oder Studienmitarbeitenden bei Ernährungsinterventionen zu verblinden, was das Risiko von Verzerrungen und Fehlern erhöht. Zudem werden die Ernährungsinterventionen oft nur sehr kurz durchgeführt, da sie beispielsweise nur während der Hospitalisation realisiert werden können oder die finanziellen Ressourcen nicht gegeben sind. Die Finanzierung ernährungsmedizinischer Studien ist im Vergleich zur pharmazeutischen Forschung oft erschwert [36]. Ein entscheidender Faktor für die Ernährungsforschung ist zudem der ethische Aspekt. Für wissenschaftlich fundierte Evidenzen benötigt es in der Regel RCT, also Studien mit einer Kontrollgruppe. Inwieweit es gerechtfertigt werden kann, Mangelernährten eine Ernährungstherapie zu verwehren (Kontrollgruppe), ist oft unklar und muss von einer Ethikkommission abgewogen und entschieden werden [28].

### Umsetzung im klinischen Alltag

Historisch bedingt hat die Ernährungsmedizin als Disziplin keinen hohen Stellenwert in der Aus- und Weiterbildung von Ärzt*innen, weswegen diese Berufsgruppe selten intrinsisches Interesse an Ernährungstherapie und der Forschung auf diesem Gebiet entwickelt [27]. Dieser Herausforderung wird nun in der Schweiz entgegengewirkt, indem die Gesellschaft für klinische Ernährung der Schweiz (GESKES) zur Verankerung der Ernährungsmedizin in der ärztlichen Aus‑, Weiter- und Fortbildung ein Weiterbildungsprogramm für den interdisziplinären Schwerpunkt „Ernährungsmedizin“ anbietet. Mit der Etablierung von Institutionen, die einen ernährungsmedizinischen Schwerpunkt aufweisen, soll die klinische Ernährung in interdisziplinären und interprofessionellen Teams gestärkt werden, wobei insbesondere die wissenschaftlich fundierte Ernährungsmedizin gefördert werden soll [37].

Außerdem haben viele Institutionen ein strukturelles Problem, da häufig gut ausgebildete Ernährungsberater*innen fehlen. In manchen Ländern werden diese zudem oft an die Küche angegliedert und nicht als Teil des Behandlungsteams gesehen bzw. eingesetzt [28]. Kosten-Nutzen-Analysen, welche die Einführung eines Mangelernährungsscreenings mit anschließender individualisierter Ernährungstherapie durch ausgebildete Fachkräfte untersuchten, konnten jedoch die Kosteneffizienz nachweisen, beispielsweise in Bezug auf erniedrigte Rehospitalisierungsraten, weniger nosokomiale Infektionen und niedrigere Sterblichkeitsrate [38]. Somit gilt es nun, die Aufmerksamkeit für das Thema Mangelernährung zu erhöhen, sowohl in den Kliniken als auch bei den zuständigen politischen Stellen.

## Fazit für die Praxis

- Mangelernährung ist häufig mit Krankheit assoziiert und multifaktoriell bedingt. Sie geht mit einem erhöhten Risiko für Funktionseinschränkungen und Rehospitalisierungen sowie mit einer erhöhten Mortalität einher.
- Mit den Kriterien der Global Leadership Initiative on Malnutrition (GLIM) stehen neue spezifischere Diagnosekriterien für die Mangelernährung zur Verfügung, die aber einer weiteren Validierung bedürfen. Eine Validierung und Optimierung dieser Kriterien ist wichtig für den klinischen Alltag, für die zukünftige Ernährungsforschung und auch für die Anerkennung bzw. finanzielle Vergütung des komplexen Syndroms.
- Neue Studien zeigen, dass ein flächendeckendes Screening für Mangelernährung bei Krankenhausaufnahme gefolgt von einer individualisierten Ernährungstherapie signifikant das klinische Outcome verbessert.
- Weitere Forschung zu Ernährungsbiomarkern ist nötig, um noch spezifischer auf die individuellen ernährungsmedizinischen Bedürfnisse von Patient*innen eingehen zu können.
- Andere zukünftige Herausforderungen bestehen im Vorantreiben der evidenzbasierten Ernährungsmedizin durch Stärkung der Ernährungsforschung sowie in der Implementierung der Erkenntnisse im klinischen Alltag und in der Ausbildung des Gesundheitspersonals.

## Footnotes

1 Beispielsweise in Literatursuche auf PubMed (25.11.2022) mit dem Suchbegriff *malnutrition[Title/Abstract]* Anstieg von 677 Publikationen in 2001 auf 4030 in 2021; mit dem Suchbegriff *clinical nutrition[Title/Abstract]* Anstieg von 23 Publikationen in 2001 auf 173 in 2021.

## References

[1] Ernährung DGf (2018). DGE-Praxiswissen: Mangelernährung in Kliniken Deutsche Gesellschaft für Ernährung e. V..

[2] Cederholm T, Barazzoni R, Austin P, Ballmer P, Biolo G, Bischoff SC (2017). ESPEN guidelines on definitions and terminology of clinical nutrition. Clin Nutr.

[3] Felder S, Braun N, Stanga Z, Kulkarni P, Faessler L, Kutz A (2016). Unraveling the link between malnutrition and adverse clinical outcomes: Association of acute and chronic malnutrition measures with blood biomarkers from different pathophysiological states. Ann Nutr Metab.

[4] Preiser JC, Ichai C, Orban JC, Groeneveld AB (2014). Metabolic response to the stress of critical illness. Br J Anaesth.

[5] Pirlich M, Schütz T, Norman K, Gastell S, Lübke HJ, Bischoff SC (2006). The German hospital malnutrition study. Clin Nutr.

[6] van Vliet IMY, Gomes-Neto AW, de Jong MFC, Jager-Wittenaar H, Navis GJ (2020). High prevalence of malnutrition both on hospital admission and predischarge. Nutrition.

[7] van Bokhorst-de van der Schueren MA, Lonterman-Monasch S, de Vries OJ, Danner SA, Kramer MH, Muller M (2013). Prevalence and determinants for malnutrition in geriatric outpatients. Clin Nutr.

[8] Besora-Moreno M, Llauradó E, Tarro L, Solà R (2020). Social and economic factors and malnutrition or the risk of malnutrition in the elderly: a systematic review and meta-analysis of observational studies. Nutrients.

[9] Pourhassan M, Sieske L, Janssen G, Babel N, Westhoff TH, Wirth R (2020). The impact of acute changes of inflammation on appetite and food intake among older hospitalised patients. Br J Nutr.

[10] Sharma K, Mogensen KM, Robinson MK (2019). Pathophysiology of critical illness and role of nutrition. Nutr Clin Pract.

[11] Felder S, Lechtenboehmer C, Bally M, Fehr R, Deiss M, Faessler L (2015). Association of nutritional risk and adverse medical outcomes across different medical inpatient populations. Nutrition.

[12] Kondrup J, Rasmussen HH, Hamberg O, Stanga Z (2003). Nutritional risk screening (NRS 2002): a new method based on an analysis of controlled clinical trials. Clin Nutr.

[13] Schuetz P, Seres D, Lobo DN, Gomes F, Kaegi-Braun N, Stanga Z (2021). Management of disease-related malnutrition for patients being treated in hospital. Lancet.

[14] Cederholm T, Jensen GL, Correia M, Gonzalez MC, Fukushima R, Higashiguchi T (2019). GLIM criteria for the diagnosis of malnutrition—A consensus report from the global clinical nutrition community. Clin Nutr.

[15] ICD-11 for Mortality and Morbidity Statistics. https://icd.who.int/browse11/l-m/en#/http%3a%2f%2fid.who.int%2ficd%2fentity%2f1153296343. Zugegriffen: 19. Mai 2023

[16] Cederholm T, Rothenberg E, Barazzoni R (2022). A clinically relevant diagnosis code for “malnutrition in adults” is needed in ICD-11. J Nutr Health Aging.

[17] Gomes F, Baumgartner A, Bounoure L, Bally M, Deutz NE, Greenwald JL (2019). Association of nutritional support with clinical outcomes among medical inpatients who are malnourished or at nutritional risk: an updated systematic review and meta-analysis. JAMA Netw Open.

[18] Deutz NE, Matheson EM, Matarese LE, Luo M, Baggs GE, Nelson JL (2016). Readmission and mortality in malnourished, older, hospitalized adults treated with a specialized oral nutritional supplement: A randomized clinical trial. Clin Nutr.

[19] Schuetz P, Fehr R, Baechli V, Geiser M, Deiss M, Gomes F (2019). Individualised nutritional support in medical inpatients at nutritional risk: a randomised clinical trial. Lancet.

[20] Gomes F, Schuetz P, Bounoure L, Austin P, Ballesteros-Pomar M, Cederholm T (2018). ESPEN guidelines on nutritional support for polymorbid internal medicine patients. Clin Nutr.

[21] Gupta RD, Ramachandran R, Venkatesan P, Anoop S, Joseph M, Thomas N (2017). Indirect calorimetry: from bench to bedside. Indian J Endocrinol Metab.

[22] Bendavid I, Lobo DN, Barazzoni R, Cederholm T, Coëffier M, de van der Schueren M (2021). The centenary of the Harris-Benedict equations: How to assess energy requirements best? Recommendations from the ESPEN expert group. Clin Nutr.

[23] Kaegi-Braun N, Germann S, Faessli M, Kilchoer F, Dragusha S, Tribolet P (2022). Effect of micronutrient supplementation in addition to nutritional therapy on clinical outcomes of medical inpatients: results of an updated systematic review and meta-analysis. Eur J Clin Nutr.

[24] Kaegi-Braun N, Tribolet P, Gomes F, Fehr R, Baechli V, Geiser M (2021). Six-month outcomes after individualized nutritional support during the hospital stay in medical patients at nutritional risk: Secondary analysis of a prospective randomized trial. Clin Nutr.

[25] Kaegi-Braun N, Kilchoer F, Dragusha S, Gressies C, Faessli M, Gomes F (2022). Nutritional support after hospital discharge improves long-term mortality in malnourished adult medical patients: Systematic review and meta-analysis. Clin Nutr.

[26] Schuetz P (2017). Food for thought: why does the medical community struggle with research about nutritional therapy in the acute care setting?. BMC Med.

[27] Crowley J, Ball L, Hiddink GJ (2019). Nutrition in medical education: a systematic review. Lancet Planet Health.

[28] Kaegi-Braun N, Baumgartner A, Gomes F, Stanga Z, Deutz NE, Schuetz P (2020). Evidence-based medical nutrition—A difficult journey, but worth the effort!. Clin Nutr.

[29] Mueller C, Compher C, Ellen DM, American Society for Parenteral and Enteral Nutrition (A.S.P.E.N.) Board of Directors (2011). A.S.P.E.N. clinical guidelines: Nutrition screening, assessment, and intervention in adults. JPEN J Parenter Enteral Nutr.

[30] Kaegi-Braun N, Mueller M, Schuetz P, Mueller B, Kutz A (2021). Evaluation of nutritional support and in-hospital mortality in patients with malnutrition. JAMA Netw Open.

[31] Radziwill R, Weimann A, Schütz T, Ohlrich-Hahn S, Fedders M, Grünewald G (2019). Überleitung vom stationären in den ambulanten Bereich. Ernährungsmedizin, Ernährungsmanagement, Ernährungstherapie : interdisziplinärer Praxisleitfaden für die klinische Ernährung.

[32] Bargetzi A, Emmenegger N, Wildisen S, Nickler M, Bargetzi L, Hersberger L (2021). Admission kidney function is a strong predictor for the response to nutritional support in patients at nutritional risk. Clin Nutr.

[33] Bonilla-Palomas JL, Gamez-Lopez AL, Castillo-Dominguez JC, Moreno-Conde M, Lopez Ibanez MC, Alhambra Exposito R (2016). Nutritional intervention in malnourished hospitalized patients with heart failure. Arch Med Res.

[34] Merker M, Felder M, Gueissaz L, Bolliger R, Tribolet P, Kagi-Braun N (2020). Association of baseline inflammation with effectiveness of nutritional support among patients with disease-related malnutrition: a secondary analysis of a randomized clinical trial. JAMA Netw Open.

[35] Casaer MP, Van den Berghe G (2014). Nutrition in the acute phase of critical illness. N Engl J Med.

[36] Davis CD, Ohlhorst S (2014). The future of nutrition research at the National Institutes of Health. Adv Nutr.

[37] Ballmer PE, Schütz P, Genton L, Stanga Z, Keller U (2021). Ernährungsmedizin, ein neuer interdisziplinärer Schwerpunkt. Schweiz Ärzteztg.

[38] Schuetz P, Sulo S, Walzer S, Vollmer L, Brunton C, Kaegi-Braun N (2021). Cost savings associated with nutritional support in medical inpatients: an economic model based on data from a systematic review of randomised trials. BMJ Open.

